# Internal-Specific Morphological Analysis of Sciatic Nerve Fibers in a Radiofrequency-Induced Animal Neuropathic Pain Model

**DOI:** 10.1371/journal.pone.0073913

**Published:** 2013-09-16

**Authors:** Samjin Choi, Hyuk Jai Choi, Youjin Cheong, Young-Jin Lim, Hun-Kuk Park

**Affiliations:** 1 Department of Biomedical Engineering and Healthcare Industry Research Institute, Kyung Hee University, Seoul, Korea; 2 Department of Neurosurgery, Hallym University, Chuncheon, Korea; 3 Department of Neurosurgery, Kyung Hee University, Seoul, Korea; 4 Department of Medical Engineering, Kyung Hee University, Seoul, Korea; National Research Council, Italy

## Abstract

This study investigated the reversible effects of pulsed radiofrequency (PRF) treatment at 42°C on the ultrastructural and biological changes in nerve and collagen fibers in the progression of neuropathic pain after rat sciatic nerve injury. Assessments of morphological changes in the extracellular matrices by atomic force microscopy and hematoxylin-eosin, Masson’s trichrome and picrosirius-red staining as well as the expressions of two fibril-forming collagens, types-I and -III, and two inflammatory cytokines, TNF-α and IL-6, were evaluated on day 30 after RF exposure. There were four groups for different RF thermal treatments: no treatment, no current, PRF, and continuous RF (CRF). An RF procedure similar to that used in human clinical trials was used in this study. The CRF treatment at 82°C led to neural and collagen damage by the permanent blockage of sensory nociceptors. The PRF treatment led to excellent performance and high expandability compared to CRF, with effects including slight damage and swelling of myelinated axons, a slightly decreased amount of collagen fibers, swelling of collagen fibril diameters, decreased immunoreactivity of collagen types-I and -III, presence of newly synthesized collagen, and recovery of inflammatory protein immunoreactivity. These evidence-based findings suggest that PRF-based pain relief is responsible for the temporary blockage of nerve signals as well as the preferential destruction of pain-related principal sensory fibers like the Aδ and C fibers. This suggestion can be supported by the interaction between the PRF-induced electromagnetic field and cell membranes; therefore, PRF treatment provides pain relief while allowing retention of some tactile sensation.

## Introduction

Peripheral neuropathic pain characterized by hyperalgesia and allodynia arises as a result of damage to the nervous system associated with inflammation [1]–[5]. There are several theories about the mechanism of neuropathic pain, including that some pro-inflammatory mediators derived from immune cells and glial cells play an important role in the pathogenesis of neuropathic pain. Damage to the peripheral nervous system leads to the activation of resident macrophages and Schwann cells, which recruit hematogenous immune cells [6]–[9]. These immune cells release several cytokines including tumor necrosis factor (TNF)-α and interleukin (IL)-1β. In addition, IL-6 is a key component of the injury response process of the nervous system and plays important roles in the neuroprotection and modulation of pain [2], [4]. However, the mechanism of action underlying neuropathic pain is poorly understood, and the selection of effective treatment methods to relieve neuropathic pain is often controversial.

Radiofrequency (RF)-induced lesions have become very popular in the treatment of pain syndromes. The effects of pain relief are essentially due to the high temperature at the tip of an electrode positioned within the neurologic tissue, which causes the coagulative necrosis of both cells and fibers [10]–[14]. Since conventional continuous RF (CRF) might be associated with severe neurodestruction, it cannot be directly applied to peripheral nerves with motor fibers [15]. CRF with electrode temperatures of 60–80°C has been generally used in clinical procedures [12], [14], [16], [17]. However, the neurolytic CRF technique using temperatures above 60°C can cause severe neurodestructive effects, and it has the potential for causing other neuropathies [12], [18]–[20]. An RF technique with lower energy or temperature is proposed in order to cause minimal tissue destruction. Therefore, the pulsed RF (PRF) procedure, in which the RF current is applied in a pulsed fashion, was introduced as an alternative method for neuropathic pain management. PRF does not cause significant sensory damage, and there is little discomfort associated with its application as compared to that of CRF [10]–[15], [21]–[25]. Superior clinical findings along with the absence of permanent neurological damage have led to the increased use of this method in the field of neuropathic pain as well as for lumbar back pain [11], [26]. Some studies [10]–[15] have compared the structural and morphological changes in the dorsal root ganglion (DRG) and the sciatic nerve after PRF or CRF treatments; however, the pathophysiologic mechanism underlying the methodological effectiveness is still not fully understood.

Peripheral nerves consist of three connective tissue sheaths, the epineurium, perineurium, and endoneurium. The epineurium is the outermost layer including the connective tissue and the blood vessels supplying the nerve. The perineurium consists of flat perineurial cells and collagen fibers (CFs). The endoneurium consists of CFs, reticular fibers, and an extracellular matrix (ECM) occupying the space between the nerve fibers (NFs) within the fascicle [27]–[31]. The CF diameter increases with the distance from the nerve center, with larger CFs found in the epineurium, and smaller CFs found in the endoneurium [30]. The endoneurial CFs form the walls of the endoneurial tubules. Axons are accompanied by Schwann cells in these tubules. There are some non-fibrous collagens in the Schwann cell basement membranes [32]. The non-cellular components of these supportive sheaths are mainly CFs, which serve as scaffolds for NFs [33]. Several collagen types have been found in peripheral nerves, including collagen types I, III, IV, V, XV and XXVIII [30], [34]–[37]. Collagen types I, III and V are fibril-forming collagens, while collagen types IV and XV are basement membrane collagens.

To the best of the authors’ knowledge, there have been no studies evaluating the crucial contribution of the ECM in the progression of RF-induced neuropathic pain in the peripheral nerves. This goal of this study was to investigate the reversible changes in the ultrastructure and biology of NFs and CFs in the progression of neuropathic pain after sciatic nerve injury induced by PRF at 42°C. Histological, immunohistochemical and atomic force microscopy (AFM) assessments were used to examine the morphological changes in *in vivo* rat sciatic nerves after RF exposure. The RF procedures used in this study were similar to methods used in human clinical trials and which are also applied to animals.

## Results

### Morphological Change in Myelinated Axons upon RF-induced Sciatic Nerve Injury

**Figure 1:**
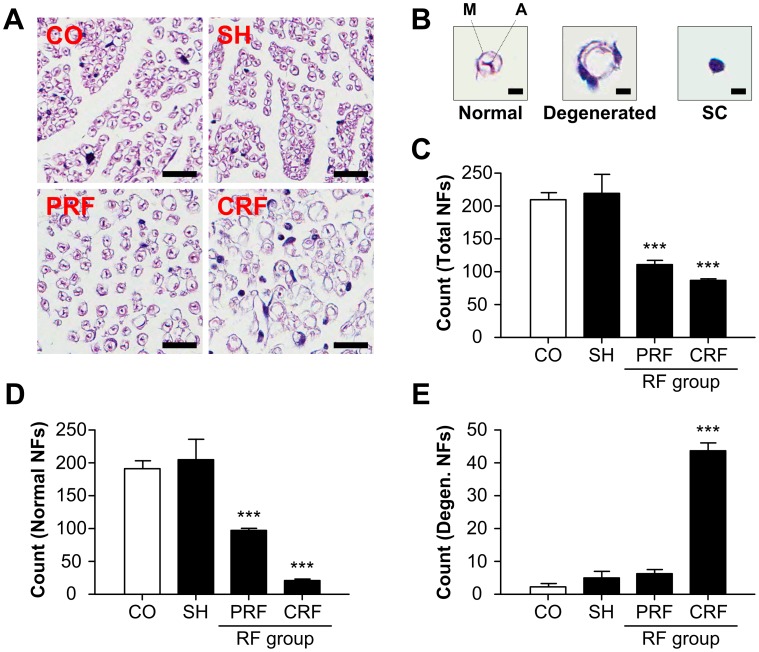
Morphological changes in NFs for RF-induced rat sciatic nerve injury. NF, nerve fiber. RF, radiofrequency. (A) The CO and SH groups exhibited normal ovoid-shaped NFs, while the PRF group exhibited some myelinated axon damage and swelling of the myelinated axons, and the CRF group had severely degenerated and stunted myelinated axons or swelling and absence of mitochondria. CO, control; SH, sham; PRF, pulsed RF; CRF, continuous RF. Scale bar = 50 µm. (B) Definition of NFs for counting. M, myelin; A, axon. Scale bar = 5 µm. (C) Each group showed significantly different numbers of total, normal and degenerated NFs (Table 1). Both RF groups showed half of the total numbers of NFs in the CO group. (D) RF led to a significant decrease in normal NFs compared to the CO group. (E) CRF led to a significant increase in degenerated NFs compared to the CO group. These analyses were performed within an area of 250×250 µm^2^. Data shown are the mean and SD of six mice (n = 18). ***P<0.0001 vs. CO.

The sciatic NFs of the rat peripheral nerves were observed on day 2 after the RF treatment by H&E staining (Fig. 1A). The control group (CO) and sham group (SH) showed normal ovoid-shaped NFs consisting of conductive axons (A) and axon-ensheathing myelins (M). The PRF group showed some myelinated axon damage and swelling of the myelinated axons. The CRF group had severely degenerated and stunted myelinated axons. The numbers of normal and total NFs for the control and sham groups were significantly larger than those for the RF groups (Fig. 1C and D and Table 1; P<0.0001 vs. CO). In addition, the numbers of degenerated NFs for the control and sham groups were significantly smaller than those for the RF groups, particularly those in the CRF group (Fig. 1E).

**Table 1:** Morphological changes in nerve fibers for RF-induced rat sciatic nerve injury (n = 18). This calculation was performed within an area of 250×250 µm^2^.

Nerve fibers	Control	Sham	PRF	CRF
Normal	192.33±10.60	205±31.48	98.33±2.52***	21.67±1.15***
Degenerated	2.33±1.15	5±2	6.33±1.15	43.67±2.52***
Schwann cell	15.33±1.15	9.67±3.06*	7±3.46*	21.33±2.08***
Total	210±10.82	219.67±30.24	111.67±6.66***	86.67±2.08***

* P<0.05,

*** P<0.0001 vs. CO.

### Change in Total CFs upon RF-induced Sciatic Nerve Injury

**Figure 2:**
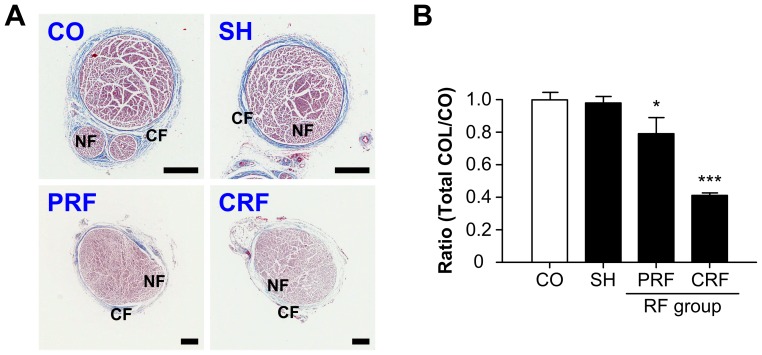
Quantitative changes in total CFs for RF-induced rat sciatic nerve injury. CF, collagen fiber. RF, radiofrequency. (A) CO and SH groups showed normal collagen structure encasing NFs while the RF groups showed significant decreases in total collagen density. CO, control; SH, sham; PRF, pulsed RF; CRF, continuous RF. CF = epi-perineurial layer; NF, nerve fiber = endoneurial layer. Scale bar = 250 µm. (B) Ratios of total collagen for each treatment group normalized against the CO group. COL, collagen. Data shown are the mean and SD of six mice (n = 18). *P<0.05; ***P<0.0001 vs. CO.

The total CFs of the rat peripheral nerve were observed on day 2 after the RF treatment by MT staining (Fig. 2A). The control group showed a normal peripheral nerve structure in which the CFs encase NFs ensheathing conductive axons. The sham group showed no significant morphological or quantitative changes in the epi-perineurial collagen. However, the RF groups showed significant decreases in total collagen density (P<0.0001 vs. CO); the PRF group showed a slightly decreased amount of CFs, and the CRF group showed complete destruction of collagen structure or dead collagenous material (Fig. 2B).

### Morphological Change in CFs on RF-induced Sciatic Nerve Injury

**Figure 3:**
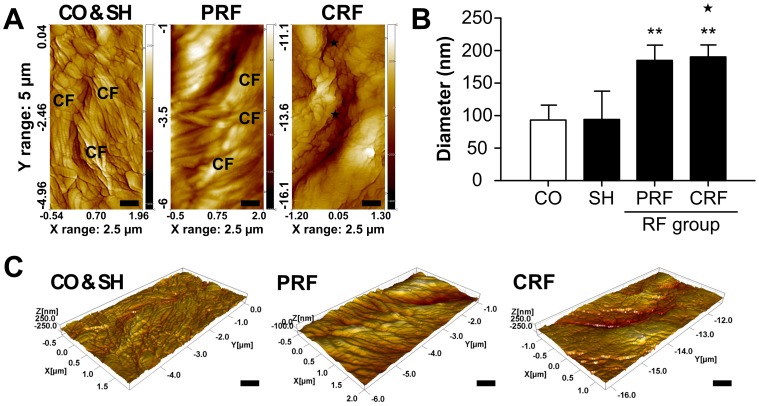
Morphological changes in the epineurial CFs for RF-induced rat sciatic nerve injury. CF, collagen fiber. RF, radiofrequency. (A) Nanostructural alternation of RF-induced CFs was evaluated by AFM throughout a postoperative period of 30 days using an NANOS N8 NEOS with a scan size of 2500 × 5000 nm^2^ and a scan speed of 0.8 lines/s at 35% relative humidity and room temperature. AFM, atomic force microscopy. CO, control; SH, sham; PRF, pulsed RF; CRF, continuous RF. ★, suspected CFs. Scale bar = 500 nm. (B) The mean diameters of the epineurial CFs for the CO and SH groups were 93.78±23.09 nm and 94.98±43.07 nm, respectively. PRF led to a significant swelling of the epineurial CFs (186.55±22.74 nm). There were no collagen composites in the CRF group, but there were some suspected CF composites (★ mark, 191.27±19.50 nm). (C) Three-dimensional AFM images were reconstructed from AFM tapping-mode topographical images by SPIP software. These images show the clear axial periodicity of the CFs. Scale bar = 500 nm. Data shown are the mean and SD of three mice (n = 9). **P<0.005 vs. CO.

The ultrastructure of CFs in the epineurium of each rat peripheral nerve was observed by AFM assessment on day 7 after the RF treatment (Fig. 3). From the AFM tapping-mode nanostructural topographical images (2500×5000 nm^2^), the normal groups (CO, SH) showed an irregular parallel arrangement of CFs with clear axial periodicity. The mean diameters of the control and sham groups were 93.78±23.09 nm and 94.98±43.07 nm with mean fibril D-periodicities of 67.63 nm and 68.25 nm, respectively. However, the RF groups showed a significant increase in the diameter of the CFs. The PRF group (186.55±22.74 nm, P<0.005 vs. CO) showed a distinctly larger mean diameter of CFs than the control and sham groups. Most CRF group images showed no collagen composites, but some suspected CF composites (★mark, 191.27±19.50 nm, P<0.005 vs. CO) were detected according to the imaging areas (Fig. 3A and B). If these composites are regarded as CFs, no significant differences in the mean D-periodicity were seen between the two RF groups. Three-dimensional (3D) AFM images show details of the complex 3D topographical organization of the nanostructural effects from the RF treatment on the epineurium of the rat peripheral nerve (Fig. 3C).

### Change in Collagen Type upon RF-induced Sciatic Nerve Injury

**Figure 4:**
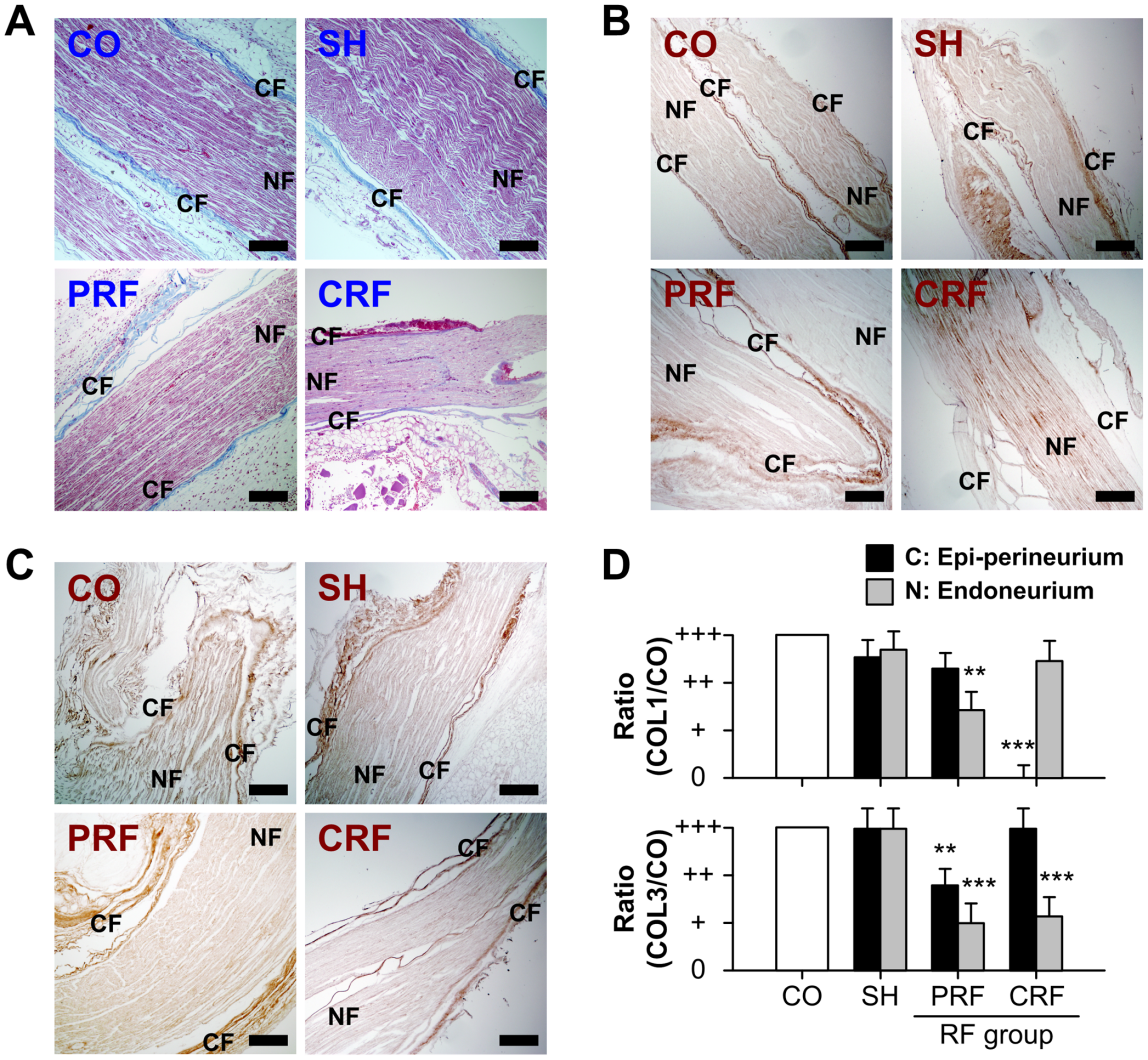
Changes in collagen type for RF-induced rat sciatic nerve injury. (A) CO, SH and PRF groups showed the normal collagen ultrastructure encasing NFs, while the CRF group showed loss or destruction of collagen structure. CO, control; SH, sham; PRF, pulsed RF; CRF, continuous RF. CF, collagen fiber = epi-perineurial layer; NF, nerve fiber = endoneurial layer. Scale bar = 200 µm. The intensities of immunoreactivity for (B) collagen type I and (C) collagen type III in the epi-perineurium and endoneurium of rat sciatic nerves on day 7 after the RF treatment were decreased in comparison with those in the CO and SH groups. Scale bar = 200 µm. (D) The PRF group showed significant decreases in the protein expression of both endoneurial collagen types I and III (P<0.005). The CRF group showed significant decreases in the protein expression of epi-perineurial collagen type I (P<0.0001) and endoneurial collagen type III (P<0.0001). Data shown are the mean and SD of six mice (n = 18). COL1, collagen type I; COL3, collagen type III. **P<0.005, ***P<0.0001 vs. CO.

The collagen types in the rat peripheral nerves were observed on day 7 after the RF treatment by MT staining and immunohistochemistry (Fig. 4). The control and sham groups showed the normal CF ultrastructure encasing NFs. The RF treatment led to the loss or destruction of collagen structure; for example (CRF panel of Fig. 4A), the upper epi-perineurial CFs were degenerated (red), and the lower epi-perineurial CFs might have been dead (light blue). The intensities of immunoreactivity for collagen type I (COL1, Fig. 4B) and collagen type III (COL3, Fig. 4C) in the collagen layers (C; epi-perineurium) and nerve layers (N; endoneurium) of the rat sciatic nerves on day 7 after the RF treatment were decreased in comparison with those in the control and sham groups. The CRF group showed significant decreases in the protein expression of epi-perineurial collagen type I (P<0.0001 vs. CO) and endoneurial collagen type III (P<0.0001 vs. CO). The PRF group showed significant decreases in protein expressions of both endoneurial collagen type I and collagen type III (P<0.005 vs. CO, Fig. 4D).

### Changes in Collagen Formation upon RF-induced Sciatic Nerve Injury

**Figure 5:**
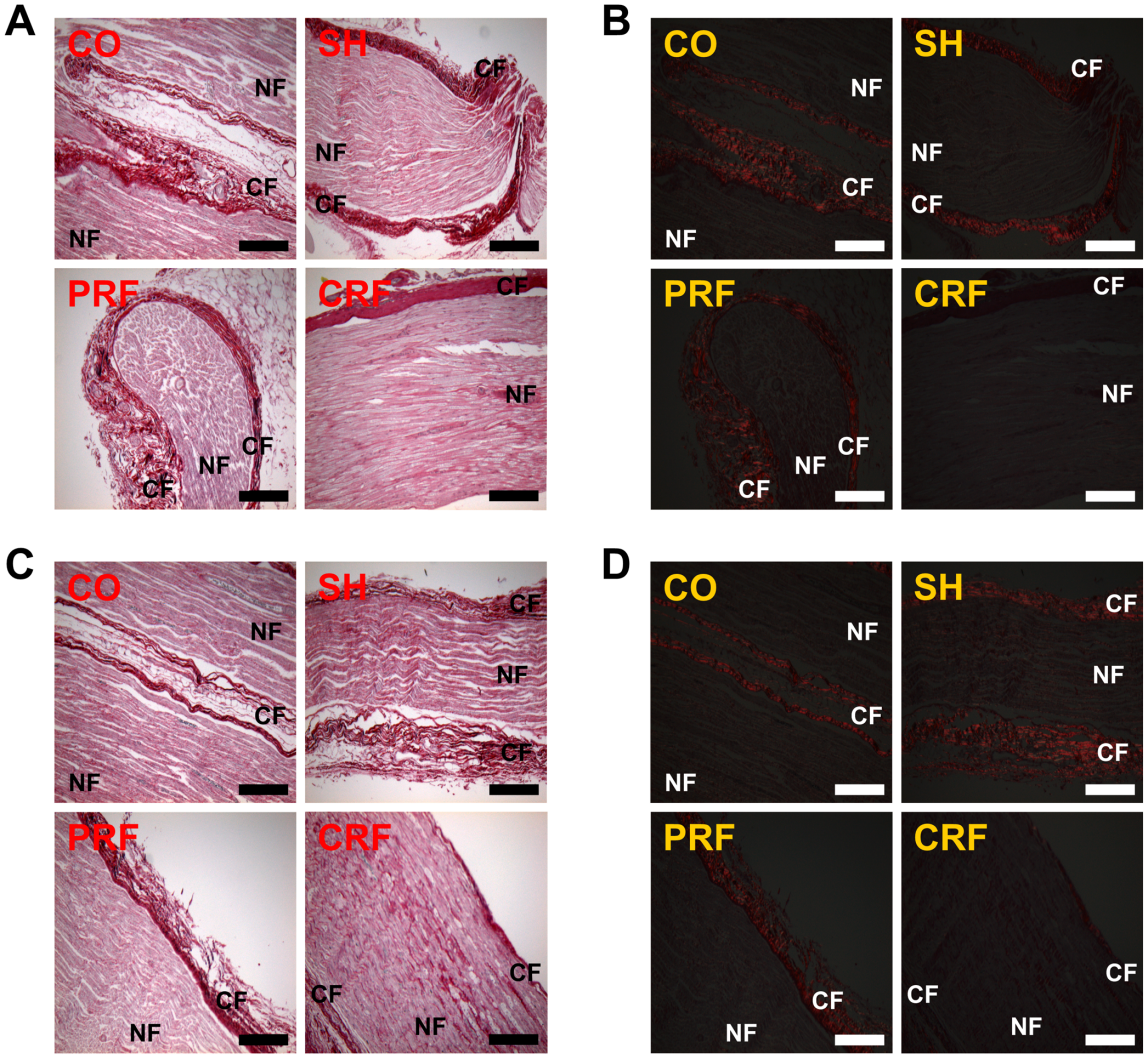
Observation of collagen formation for rat sciatic nerve injury immediately (A, B) and on day 7 (C, D) after RF treatment under bright fields and polarized fields based on PR staining. RF, radiofrequency. PR, picrosirius red (collagen). (A) PR images of the sciatic nerve under bright field did not yield information about the degree of degeneration or destruction of CFs or NFs immediately after RF treatment. CO, control; SH, sham; PRF, pulsed RF; CRF, continuous RF. CF, collagen fiber = epi-perineurial layer; NF, nerve fiber = endoneurial layer. Scale bar = 200 µm. (B) PR images under polarized field clearly showed the formation of newly synthesized collagen (presence of yellow-orange birefringence). Scale bar = 200 µm. (C) PR images of the sciatic nerve under bright field on day 7 after RF treatment. Scale bar = 200 µm. (D) Newly synthesized collagen formation was examined in all groups under polarized field. Scale bar = 200 µm. Data shown are the mean and SD of six mice (n = 18).

The collagen formation in the rat peripheral nerve was observed by PR staining under bright fields and polarized fields immediately and on day 7 after RF treatment (Fig. 5). Immediately after the RF treatment, the PR images under the conventional bright field did not yield information about the degree of degeneration or destruction of the CFs or NFs (Fig. 5A). However, the PR images under the polarized field clearly show the formation of newly synthesized collagen, as reflected by the presence of yellow-orange birefringence, in the control, sham and the PRF groups (Fig. 5B), while there are no collagen components in the CRF group. On day 7 after the RF treatment, the newly synthesized collagen formation was examined in all groups (Fig. 5C and D).

### Changes in Inflammation upon RF-induced Sciatic Nerve Injury

**Figure 6:**
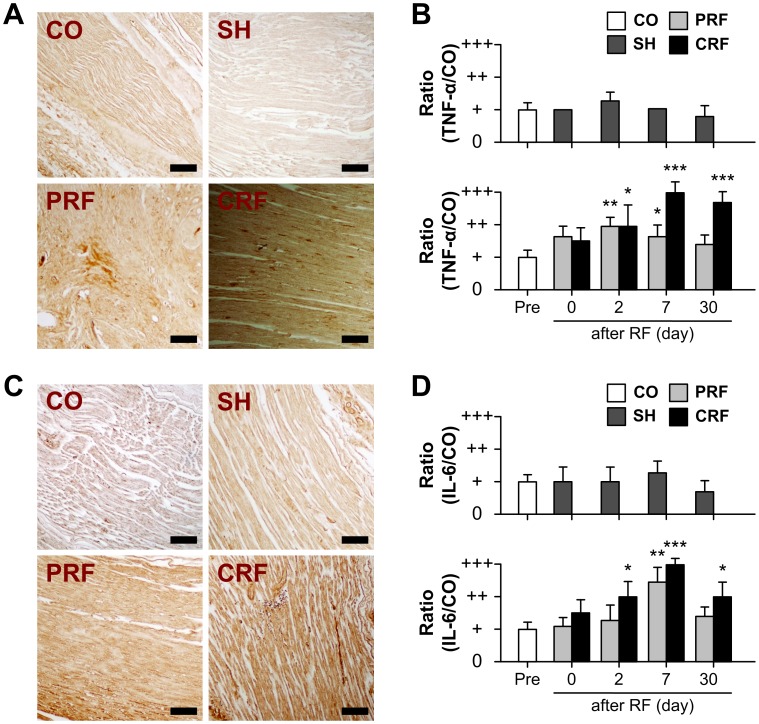
Up-regulation of TNF-α and IL-6 for RF-induced peripheral nerve injury. RF, radiofrequency. The expression of TNF-α and IL-6 proteins in rat sciatic nerves with no treatment (CO), no current (SH), PRF and CRF was visualized by immunohistochemistry before stimulation (Pre) and on days 0, 2, 7 and 30 after stimulation. (A) Representative immunohistochemistry images on day 7 after RF treatment are shown. CO, control; SH, sham; PRF, pulsed RF; CRF, continuous RF. Scale bar = 100 µm. (B) The SH group showed no significant changes in TNF-α expression over the 30-day experimental period as compared to the CO group. In contrast, TNF-α expression was up-regulated immediately after RF treatment. This up-regulation persisted until days 7 and 30 after the PRF and CRF treatments, respectively. (C) Changes in IL-6 expression were similar to those observed for TNF-α. Scale bar = 100 µm. (D) IL-6 immunoreactivity reached a maximum on day 7 after exposure to PRF (P<0.005) and CRF (P<0.0001). Data shown are the mean and SD of six mice (n = 18). *P<0.05, **P<0.005, ***P<0.0001 vs. CO.

The immunoreactivity of two inflammatory proteins was observed in the rat peripheral nerve up to day 30 after the RF treatment (Fig. 6). Overall, changes in TNF-α expression were similar to those observed for IL-6. The control and sham groups showed no significant changes in TNF-α or IL-6 expression over the 30-day experimental period. TNF-α and IL-6 expressions were up-regulated immediately after RF treatment in the PRF and CRF groups. This up-regulation persisted until days 7 and 30 after the PRF and CRF treatments, respectively. TNF-α immunoreactivity reached a maximum on days 2 and 7 after exposure to PRF (P<0.005 vs. CO) and CRF (P<0.0001 vs. CO), respectively. IL-6 immunoreactivity reached a maximum on day 7 after exposure to both PRF (P<0.005 vs. CO) and CRF (P<0.0001 vs. CO).

## Discussion

In this study, we demonstrated the crucial role of PRF treatment, which causes reversible change in NFs and CFs, in the development of neuropathic pain after peripheral nerve injury. It is accepted that the CRF technique was developed for the treatment of chronic pain syndrome in clinical practice. The mechanism of pain relief by CRF is due to the high temperature surrounding an electrode placed in the tissue, which blocks the nerves from transmitting pain signals. CRF treatment with electrode temperatures of 60–80°C has generally been used in clinical procedures [14], [16], [17]. However, since the CRF procedure using temperatures above 60°C can cause severe neurodestructive effects, it has the potential to cause other neuropathies [12], [18]–[20]. Therefore, CRF has not been directly applied to peripheral nerves with motor fibers [15]. An RF technique with lower energy (50%) or low temperature (42°C) is proposed to minimize tissue destruction. PRF does not cause significant sensory damage, and there is little discomfort associated with its application compared to CRF [10]–[15], [21]–[25]. Superior clinical results along with the absence of permanent neurological damage have led to the increased use of this method in the field of neuropathic pain as well as for lumbar back pain [11], [26]. Although several studies [10]–[15] have compared structural and morphological changes in the DRG and sciatic nerve after PRF or CRF treatments, the pathophysiological mechanism underlying PRF effectiveness is still not fully understood. To the best of the authors’ knowledge, there are no studies evaluating the role of the ECM in the progression of neuropathic pain after the RF treatment. In this study, we demonstrated changes in the ultrastructure and biology of the NFs and CFs in the progression of neuropathic pain after RF-induced sciatic nerve injury. Overall, our data showed that PRF led to non-destructive neural and collagen damage including some myelinated axon damage and swelling of myelinated axons (Fig. 1), a slightly decreased amount of CFs (Figs. 2 and 4), swelling of CF diameters (Fig. 3), a slight decrease in the immunoreactivities of collagen types I and III (Fig. 4), the presence of newly synthesized collagen (Fig. 5), and the reversible up-regulation of inflammatory proteins (Fig. 6). Since the mechanism of pain relief by CRF treatment at 60–80°C is considered to be clearly different from that of PRF treatment at 42°C, the goal of this study was to estimate the role of PRF rather than comparing the effects of the PRF and CRF techniques on the ECM formation and inflammatory changes during the progression of neuropathic pain. The two RF procedures used in this study were similar to methods used in human clinical trials and that are also applied to animals.

In this study, we investigated the effects of RF treatment on the structure of CFs and NFs in the rat sciatic nerve. Myers et al. [32] assessed human neurological disorders associated with peripheral nerve injury by estimating the total weight of collagen in each anatomical compartment in the rat sciatic nerve and human sural nerve. The percentages of total nerve collagen in each layer of the rat sciatic nerve were 8% (63 µg/cm^2^), 43% (318 µg/cm^2^), and 49% (389 µg/cm^2^) for the epineurium, perineurium and endoneurium, respectively. Myers et al. stated that, since the separating plane between the epineurium and perineurium was often unclear, their estimation was imprecise. This study also estimated the amount of total CFs in the epi-perineurium of the rat sciatic nerve after the RF treatments (Fig. 2). The high-temperature CRF procedure resulted in the complete destruction of collagen in the epi-perineurium, a decrease of 58% (P<0.0001 vs. CO), but PRF treatment resulted in a slight decrease of 21% (P<0.05 vs. CO) in the amount of collagen in the epi-perineurium. This finding suggests that CRF led to the complete destruction of the collagen in the rat sciatic nerve and loss of its function, while PRF led to the partial destruction of the collagen and preservation of its function. This suggestion is also supported by the results of the PR histological images under polarized field; the PRF group showed the presence of newly synthesized collagen, while the CRF group showed its absence (Fig. 5). The estimation of collagen by direct chemical analysis can suggest quantitative information to support this interpretation. Therefore, biochemical and pathological studies [32] of collagen in the sciatic nerve by Raman spectroscopy and inductively coupled plasma atomic emission spectrometer are required for further study.

The morphology of collagens might be influenced by various *in vivo* collagen metabolism rates [38]. Several collagen types have been found to be expressed in peripheral nerves, including types I, III, IV, V, XV and XXVIII [30], [34]–[37]. Collagen types I, III and V are fibril-forming collagens, while collagen types IV and XV are basement membrane collagens. Collagen type XXVIII is an atypical type in the peripheral nervous system [35], [39]. To the best of the authors’ knowledge, there are no studies correlating collagen type with collagen diameter in peripheral nerves, except in diabetic neuropathy [30], [40], [41]. Wang et al. [30] showed the presence of larger CF diameters in diabetic sciatic nerves. Bradley [41] reported that collagen types I and III are more prominent in human diabetic neuropathy due to glucose concentration-dependent collagen gene up-regulation as well as increased resistance to proteolysis. The present study (Fig. 4) showed that the control and sham groups had normal CF ultrastructure encasing NFs and exhibited no changes in collagen types up to day 30 after the RF treatment. CRF led to degenerated or dead CFs, the absence of epi-perineurial collagen type I (P<0.0001 vs. CO), no significant decreases in endoneurial collagen type I or epi-perineurial collagen type III, and a significant decrease in endoneurial collagen type III (P<0.0001 vs. CO). Interestingly, PRF led to no loss of collagen structure and to decreases in the protein expression of collagen types I and III in all layers, particularly to significant decreases in the endoneurial collagen types I (P<0.005 vs. CO) and III (P<0.0001 vs. CO). No changes in collagen type I in the endoneurial layer or collagen type III in the epi-perineurial layer for the CRF group were caused by the degenerated collagens (Fig. 5). These results can be explained by CRF treatment at 82°C resulting in neuroablative or neurodestructive effects on rat sciatic nerve. PRF treatment at 42°C leads to larger decreases in the protein immunoreactivity of collagen types I and III in the endoneurium than in the epi-perineurium, which may be explained by the heat-mediated selectively neurodestructive effects on pain-related sensory fibers (Aδ and C) in the rat sciatic nerve.

The peripheral nerves contain several types of NFs including afferent sensory fibers (Aα, Aβ, Aδ and C fibers) and efferent motor fibers (Aα, Aγ, B and C fibers) with diameters of 0.1–22 µm. The majority of fibers are less than 2 µm in diameter. These fibers are mostly unmyelinated fibers, the C fibers, with diameters of 0.1–1.3 µm. There is also a smaller group of fibers with diameters of 1–4 µm (Aδ fibers) as well as additional groups with diameters of 6–12 µm (Aβ fibers) and 12–22 µm (Aα fibers). Sensory fibers convey various sensory inputs such as mechanical, thermal and noxious stimuli, while motor fibers can be divided into α-motor fibers (Aα fibers) that innervate skeletal extrafusal muscle fibers and γ-motor fibers (Aγ fibers) that innervate the spindle muscle fibers. The threshold of a fiber to externally applied electrical shocks is inversely related to the diameter of that fiber; the Aα fibers have the lowest electrical threshold, the Aδ fibers have a slightly higher threshold, and the C-fibers have the highest threshold. The A and C fibers consist of myelinated and unmyelinated fibers, respectively. The A and B fibers are most sensitive to pressure block and hypoxic block, respectively. The C fibers are most susceptible to anesthetic block. The Aδ and C fibers are the principal sensory nociceptors. This study showed that the control and sham groups had normal ovoid-shaped NFs consisting of conductive axons and axon-ensheathing myelins with various diameters. The CRF group had severely degenerated and stunted myelinated axons, and the PRF group had some myelinated axon damage and swelling of myelinated axons (Fig. 1). These findings are consistent with those of Erdine et al. [10]. PRF led to greater damage for smaller diameter fibers; the Aδ and C fibers showed greater damage than the Aβ fibers. It is thought that, since the pain-related principal sensory fibers are smaller in diameter than other non-pain-related fibers, they may be preferentially blocked or destroyed. Therefore, this effect of PRF on the Aδ and C fibers may explain the pain relief associated with the retention of some tactile sensations.

Despite much evidence that neuropathic pain is caused by lesions in the nervous system directly associated with inflammation, there are no reports showing the contribution of inflammatory-related proteins to the pathogenesis of neuropathic pain caused by RF treatment. It is considered that nerve injury triggers the activation of immune and Schwann cells, and pro-inflammatory mediators released from these cells recruit hematogenous immune cells, which exacerbate nerve inflammation [6], [7], [9]. Schwann cells produce several pro-inflammatory cytokines such as IL-1β, TNF-α, and IL-6, which are involved in mediating neuropathic pain [4], [42]. These pro-inflammatory mediators create a cytokine network that results in the chronic inflammation associated with neuropathic pain [1]–[4], [6]–[8]. These pro-inflammatory cytokines induce cyclooxygenase II (COX-2) expression in neuronal and non-neuronal cells in the DRG [3]. Thus, individual cytokines could independently trigger a transduction cascade that enhances the expression of COX-2 or inducible nitric oxide synthase (iNOS) in sensory neurons [3], [43]. The synthesis of chemokines was increased, resulting in a simultaneous increase in COX-2 or iNOS expression. Recently, it has been reported that monocyte chemoattractant protein-1 and macrophage inflammatory protein-1α contribute to Wallerian degeneration after nerve injury and are important regulators of inflammatory pain and hyperalgesia [6], [36], [44]. Therefore, in this study, progressive TNF-α and IL-6 immunoreactivities were investigated on day 30 after RF treatment (Fig. 6). There were no changes in these representative pro-inflammatory cytokines in the sham group or up-regulation of them in the RF groups as compared to the control group. The CRF-induced inflammatory cytokines showed significant up-regulation on day 2 after RF treatment that steadily increased until day 30 (P<0.05). PRF led to an increase in TNF-α and IL-6 immunoreactivities on days 2 and 7 after RF treatment that recovered to normal levels by day 30. This finding suggests that the neuropathic pain caused by RF is responsible for triggering pro-inflammatory signaling.

Similar to the PRF technique for the treatment of neuropathic pain, our group has performed the RF tissue-tightening application in ophthalmic plastic and reconstructive surgery [45], [46]. The basic principle of this method is that RF energy causes CFs to contract immediately, and then this tissue contraction and heat-mediated healing response induces the production of new collagen. We reported that, immediately after RF treatment, increases in the diameter and D-periodicity of dermal CFs were evident. Then, the diameters of the dermal CFs decreased over seven postoperative days, followed by the recovery of the decreased diameters to near baseline during the 30 postoperative days. However, the present study found that CFs exposed to PRF showed a swelling of the collagen network structure at the nanostructural level over seven postoperative days. Those injured by CRF lost their cylindrical shape and exhibited severe deformation of the collagen network structure (Fig. 3). The mechanism of PRF may be different from that of the RF tissue-tightening treatment that leads to gradual skin tightening and laxity reduction. In this study, the control and sham groups had myelinated axons with normal ultrastructures. CRF caused irreversible degenerative ultrastructural changes in NFs. These changes included the collapse of ovoid-shaped myelinated axons, the absence of a cytoskeleton, and minor swelling of mitochondria (not shown), resulting in the coagulative necrosis and Wallerian degeneration associated with chronic neuropathic pain. In contrast, PRF resulted in mild axonal damage and minor swelling of mitochondria (not shown). Tun et al. [47] observed mitochondrial edema in both PRF and CRF groups. Tun et al. [12] and Erdine et al. [15] showed the degeneration of mitochondria in only the CRF group. Sluijter et al. [25] proposed that the electromagnetic field (EMF) produced during the application of PRF is responsible for pain relief, although PRF at 42°C results in minimal tissue destruction. Li et al. [48] reported that the irreversible electroporation (IRE) method with a sequence of 10 square pulses of 3800 V/cm, each 100 ms long, led to the restoration of nerve conduction velocity, the regeneration of a large number of NFs, and the restoration of most of the myelin sheath structure at 7–10 weeks after injury. They stated this effect is responsible for IRE-induced EMF formation. In the present study, PRF treatment at 42°C had little effect on the morphology or function of the sciatic nerve; therefore, we suggest that the therapeutic effect of PRF-induced pain relief was not from tissue heating, but from PRF-induced EMF formation. In addition, the understanding and verification of electromagnetic or thermal field effects in the sciatic nerve by finite element calculation [49] is also required for further study.

Finally, we examined the morphological changes in CFs and NFs of rat sciatic nerves using eight investigation methods. H&E staining was used to examine the morphological effects of the PRF treatment on the peripheral nerves (Fig. 1). MT staining (Figs. 2 and 4) and PR staining (Fig. 5) were used to examine the quantitative changes in collagens caused by PRF on the peripheral nerves. Collagen types I and III immunoreactivities were used to examine the inflammatory response to PRF treatment on the peripheral nerves. Immunohistochemical analysis was performed for collagen types I and III to quantify the RF-induced CF alternation (Fig. 4) and for TNF-α and IL-6 to evaluate the RF-induced inflammatory responses (Fig. 6). AFM analysis to quantitatively examine the nanostructural characteristics of CFs in rat sciatic nerves treated with PRF was used for comparison with the conventional SEM analysis reported previously. This study showed that the mean diameters of the normal epineurial CFs in the rat sciatic nerves were 93.78±23.09 nm for the control group and 94.98±43.07 nm for the sham group. This morphological finding is similar to the diameter (104±37 nm) reported by Wang et al. [30]. Histopathology and AFM provided satisfactory results, as expected. In particular, AFM analysis with the force distance curve is reliable and can provide very promising results in the field of neuropathology.

In summary, the mechanism of pain relief induced by CRF treatment at 82°C is due to destructive neural and collagen damage and the permanent blockage of sensory nociceptors through other nerve pathways. This neurolytic CRF technique could result in severe neurodestructive effects, and there is high probability of causing additional neuropathies. However, PRF treatment at 42°C showed excellent performance and high expandability in all aspects compared to the CRF treatment; effects included the slight damage and swelling of myelinated axons, a slightly decreased amount of CFs, swelling of CF diameters, a slightly decreased immunoreactivity of collagen types I and III, the presence of newly synthesized collagen, and the recovery of inflammatory protein immunoreactivities. We suggest that the mechanism of PRF-induced pain relief may be due to the temporary blockage of nerve signals through pathways responsible for reversible neuronal depression as well as the preferential destruction of pain-related principal sensory fibers like the Aδ and C fibers. This suggestion can be explained by the interaction between PRF-induced EMF and the cell membranes. Thus, PRF treatment provides pain relief while allowing retention of some tactile sensation.

## Methods

### Animals

Male Sprague-Dawley rats (200–250 g) were used in the experiments. The rats were housed in a controlled environment with temperatures of 22–24°C and a 12-hr light/dark cycle and fed commercial rat chow and water *ad libitum*. Adequate injection and anesthesia were performed to minimize pain or discomfort. All animal use procedures were approved by the Ethical Committee of Kyung Hee University College of Medicine (KHMC-IACUC11-020) and were in strict accordance with the National Institutes of Health Guide for the Care and Use of Laboratory Animals.

### RF Surgery

Animals were anesthetized with an intraperitoneal injection of 350 mg/kg chloral hydrate. Additional chloral hydrate was properly administered where needed in order to maintain the same level of anesthesia during the experiment. Sciatic nerve lesions were induced in rats by RF using a NeuroTherm NT1000 (NeuroTherm Inc., Middleton, MA, USA) and designed to mimic clinical applications. A disposable 22-gauge, 5-cm-long RF cannula (S-505; NeuroTherm Inc.) with a 5-mm active tip was inserted. The introducer needle was withdrawn. The non-insulated cylindrical wall of a disposable RF electrode (RFDE-5; NeuroTherm Inc.) was advanced with light contact along the sciatic nerve, while the sharpened point or the sharp bevel edges of the electrode tip was not contacted with the sciatic nerve [10]. Then, the impedance was measured, and electrical stimulation was started one the impendence was in the proper range. This was accomplished without bleeding. According to clinical procedures, the PRF procedure was set to deliver an RF current at 42°C for 120 s with a total of 240 pulses. The CRF procedure was set to deliver an RF current at 82°C for 60 s. In the sham-treated sciatic nerves, an identical electrode placement was performed, but no RF current was applied. The animals were divided into four experimental groups of six individuals each, as follows: normal rats (control group), rats that received electrode placement without RF current (sham group), PRF-treated rats (PRF group), and CRF-treated rats (CRF group).

### Histology

The isolated sciatic nerve tissues were fixed in 10% buffered formalin, embedded in paraffin, sectioned into sections 5 µm thick, and stained with hematoxylin and eosin (H&E; inflammation), Masson’s trichrome (MT; collagen) and picrosirius red (PR; collagen). Histological assessment of the RF-induced sciatic nerve injury was performed by two pathologists blinded to the group assignments of the rats. The H&E and MT images were captured by an Olympus BX51 microscope equipped with a CCD camera (DP70; Olympus, Tokyo, Japan). PR images were captured by a microscope with filters to provide circularly polarized illumination (OSS-400PB, OSUNHITECH Co., LTD., Goyang, Korea) and equipped with a CCD camera (QICAM; Qimaging, Surrey, BC, Canada). Density-based analysis was performed using ImageJ software.

### Immunohistochemistry

Immunohistochemical analysis was performed for TNF-α and IL-6 to evaluate the RF-induced inflammatory reaction and for collagen types I and III to quantify the RF-induced CF alternation. Formalin-fixed, paraffin-embedded tissue slides were heated at 60°C for 30 to 60 min. Tissues were then deparaffinized in 100% xylene (3×3 times), 100% ethanol (3×3 times), 95% ethanol (3×3 times), 75% ethanol (3×3 times), and distilled water (H_2_O) (5 min). Antigen retrieval was performed by the microwave method in 0.1 mol/L sodium citrate (pH 6.0) for 5 min. To quench endogenous peroxidase activity, tissues were incubated with 3% hydrogen peroxide in deionized water for 10 min at room temperature (RT). Endogenous biotin activity was blocked using an avidin/biotin blocking kit (SP-2001; Vector Laboratories, Burlingame, CA, USA). Sections were then blocked for 60 min at RT in blocking buffer (5% normal goat serum, 1% bovine serum albumin, and 0.02% Triton X-100 in phosphate buffered saline [PBS]). Tissues were incubated with antibodies to TNF-α (1∶100; Santa Cruz Biotechnology, Santa Cruz, CA, USA), IL-6 (1∶100; Santa Cruz Biotechnology), collagen type I (1∶400; Santa Cruz Biotechnology), and collagen type III (1∶600; ab6310; Abcam, Cambridge, MA, USA) overnight at 4°C. To examine the expression of these proteins, tissues were washed in PBS for 30 min and then reacted with biotinylated anti-mouse or anti-rabbit IgG (1∶200; Vector Laboratories) for 60 min at RT, followed by incubation with ABC reagent (PK-6100/6101; Vector Laboratories) for 30 min at RT. Sections were stained using the DAB Chromogen/Substrate Kit (SK-4100; Vector Laboratories) for 2–5 min. Images were captured by an Olympus BX51 microscope equipped with a CCD camera (DP70; Olympus). Density-based analysis was also performed using ImageJ software.

### AFM Imaging

AFM tapping-mode topographical images were obtained throughout a postoperative period of 30 days using an NANOS N8 NEOS (Bruker, Herzogenrath, Germany) equipped with a 42.5 × 42.5 × 4 µm^3^ XYZ scanner and two Zeiss optical microscopes (Epiplan 200×/500×). The surface of each sciatic nerve was scanned in air with a scanning area of 2.5×5 µm^2^ and a scan speed of 0.8 lines/s. AFM tapping-mode imaging was performed with 35% relative humidity at RT using the silicon cantilever with an integral pyramidal shaped tip (SICONG; Santa Clara, CA, USA). The nominal tip radius and height were <10 nm and 12–16 µm, respectively. Five representative microscopic topographical images per time point for each group were used to assess the degree of collagen destruction and were measured by two observers blinded to experimental group assignment using the Scanning Probe Image Processor version 4.8 (SPIP, Image Metrology, Lyngby, Denmark).

### Statistics

Quantitative data are expressed as the mean ± standard deviation (SD). Statistical analysis was performed using a two-tailed Student’s t-test to compare the mean values obtained from two groups. P-values less than 0.05 were considered statistically significant.
